# PREDICTORS OF DEPRESSION IN KOREAN OLDER ADULT SPOUSAL CAREGIVERS: 2017 NATIONAL SURVEY OF OLDER KOREANS

**DOI:** 10.1093/geroni/igad104.2223

**Published:** 2023-12-21

**Authors:** Stacy Yun, Sara Qualls

**Affiliations:** VA Palo Alto Health Care System, Palo Alto, California, United States; University of Colorado Colorado Springs, Colorado Springs, Colorado, United States

## Abstract

Based on the sociocultural stress coping model (Knight & Sayegh, 2010), this study was aimed at examine how factors such as familism, caregiver burden, social support, and coping strategies impact Korean older adult spousal caregivers’ depression. 369 caregiver (aged 69 to 96; M = 80.68) and care recipient (aged 69 to 102; M = 81.86) spouse dyads from the 2017 National Survey of Older Koreans was used for analysis. The model demonstrated excellent model fit indices, χ2(15) = 16.29, p = .36, model χ2 to df ratio = 1.09, TLI = .99, CFI = .99, RMSEA = .02 (95% CI: .00 to .05), and SRMR = .02. Higher levels of depression was significantly predicted by lower perceived social support, β = -.30, Z = -6.35, p < .001, being older, β = .16, Z = 3.12, p = .002, less education, β = -.18, Z = -3.81, p < .001, and having fewer living family members, β= -.12, Z = -2.29, p = .02. Additionally, the relationship between caregiver burden and depression was fully mediated by perceived social support (β = .05, t = 3.23; 95% CI: .02 to .09). Results suggest that perceived social support is a salient factor related to depression experienced by older spousal caregivers in Korea. Findings imply the need for clinicians to devise creative interventions to increase caregiver’s sense of social support, particularly those who may reside in rural areas of Korea who are primarily older, received less education, and have fewer living family members.

